# Exposure of aquatic organisms to natural radionuclides in irrigation drains, Qena, Egypt

**DOI:** 10.1038/s41598-023-27594-4

**Published:** 2023-01-09

**Authors:** K. Salahel Din, N. K. Ahmed, A. Abbady, F. M. Abdallah

**Affiliations:** 1 Physics Department, Faculty of Science, South Valley University, Qena, 83523 Egypt; 2 Health Ministry Labs, Qena Branch, Egypt

**Keywords:** Ecology, Environmental sciences, Natural hazards

## Abstract

Natural radioactivity in irrigation drains was measured by gamma spectrometry, and the resulting dose rates received by aquatic organisms were estimated. Irrigation water and sediment samples were collected from 5 irrigation drains located in Qena governorate, south of Egypt. The average activity concentrations (Bq L^−1^) of ^226^Ra, ^232^Th, and ^40^ K in irrigation water were 0.76 ± 0.06, 0.27 ± 0.02, and 8.14 ± 0.71, while in sediment (Bq kg^−1^) were 24.46 ± 1.84, 20.72 ± 1.45, and 453.00 ± 28.14, respectively. The total dose rate per aquatic organism ranged from 1.94 × 10^–04^ µGy h^−1^ in Mollusc to 7.15 × 10^–04^ µGy h^−1^ in phytoplankton. These values are far from the international recommended limit 400 µGy h^−1^ for chronic exposure to aquatic organisms, and the dose rate screening value of 10 µGy h^−1^ suggested by ERICA tool. Based on these results, it is unlikely that harmful effects will appear on the considered aquatic organisms due to exposure to natural radioactivity in the studied environment.

## Introduction

The primordial radionuclides uranium, thorium, and potassium-40 are widely distributed in our environment and represent the main source of background radiation to which all living organisms are exposed. Exposure to background radiation is an ongoing and inescapable feature of life on earth. The United Nations Scientific Committee on the Effects of Atomic Radiation (UNSCEAR) reported that uranium, thorium, and potassium in soil contribute 25%, 40% and 35% of the dose received by humans^1^. The environmental behavior of these radionuclides depends to a large extent on the characteristics of the ecosystem, so understanding the behavior, mobility, and potential hazard of natural radionuclides is very important for decision-making to protect the environment.

Exposure of non-human organisms to high levels of radiation leads to the emergence of biological responses to them. Therefore, estimating the absorbed dose of those organisms is considered necessary to assess the potential effects of radiation exposure and the harmful risks that it entails in addition to its importance for setting environmental protection criteria. Many international organizations such as European Commission (EC), International Atomic Energy Agency (IAEA), International Commission on Radiological Protection (ICRP), UNSCEAR, and United States Department of Energy (US-DOE) are concerned with protecting the environment from the harmful effects of ionizing radiation and are working on setting criteria for the protection of the environment, some of which are directed to protect non-human species^2^.

Dose limits for various terrestrial and aquatic plants and animals are based on monitoring disease and mortality rates. ICRP believes that if humans are protected (not exceeding the dose limit of 1 mSv y^−1^), the non-human species will also be adequately protected^3^. IAEA and UNSCEAR have proposed a value of 1 mGy d^−1^ as dose rate limit to protect non-human species^4,5^. The dose limits set by the US-DOE are 1 mGy d^−1^ for terrestrial animals and 10 mGy d^−1^ for terrestrial plants and aquatic animals^6^.

Despite the possibility of estimating the doses to which humans are exposed from natural background radiation, the matter for non-human species is different due to the different composition and behavior of these organisms. Therefore, estimating the effects of radiation on these species is very difficult, as it is impossible to take into account all the animals and plants that are located in a particular geographical area. To overcome this, a group of organisms representing the ecosystem is selected, called reference organisms, for which models are built to help in calculating dose rates for them.

In Egypt, various studies dealing with radiological risk due to naturally occurring radionuclides in terrestrial and aquatic ecosystems focus on the exposure to humans, while exposure to non-human biota is not covered. Only study conducted by Tawfik et al. aimed to predicate radiological exposure levels of marine biota on the Mediterranean coast was found in literature^7^. Therefore, the current study aims to evaluate the natural radioactivity levels in sediments and water of irrigation drains located in Qena region, southern Egypt, which is directly affected by the fertilizers used for agricultural purposes that represent a source of freshwater pollution. In addition to calculating the external and internal dose rates for some aquatic reference organisms (*Phytoplankton*, *Molluscs*, and *Crustaceans*) depending on the measured activity in the environmental medium (sediments and water). This study will be the first effort to estimate the dose rates to aquatic organisms in the freshwater ecosystem in Egypt.

## Materials and methods

### Samples collection and preparation

**Figure 1:**
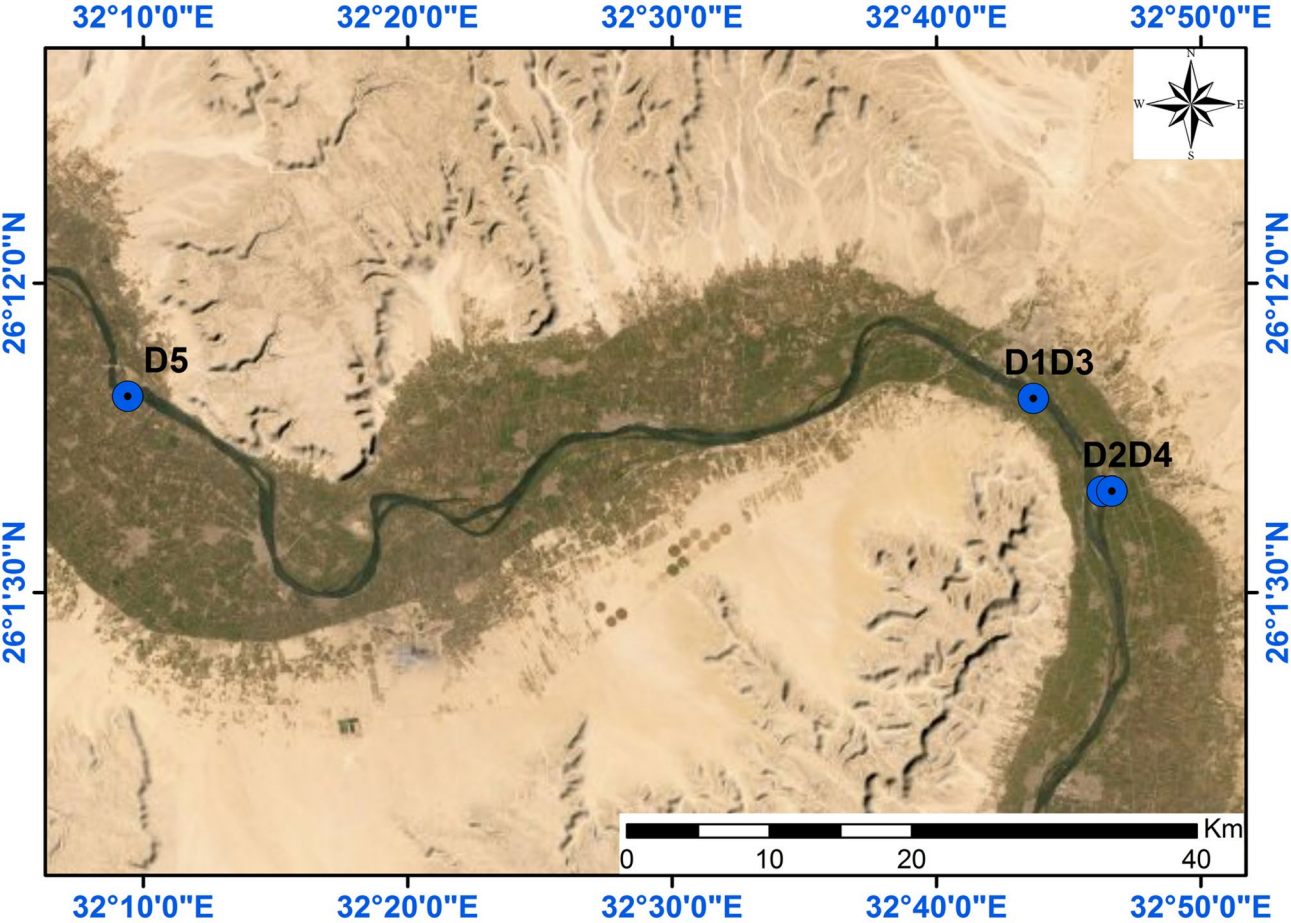
Location map of the area under study (ArcGIS software 10.8.1; ArcGIS Online).

**Figure 2:**
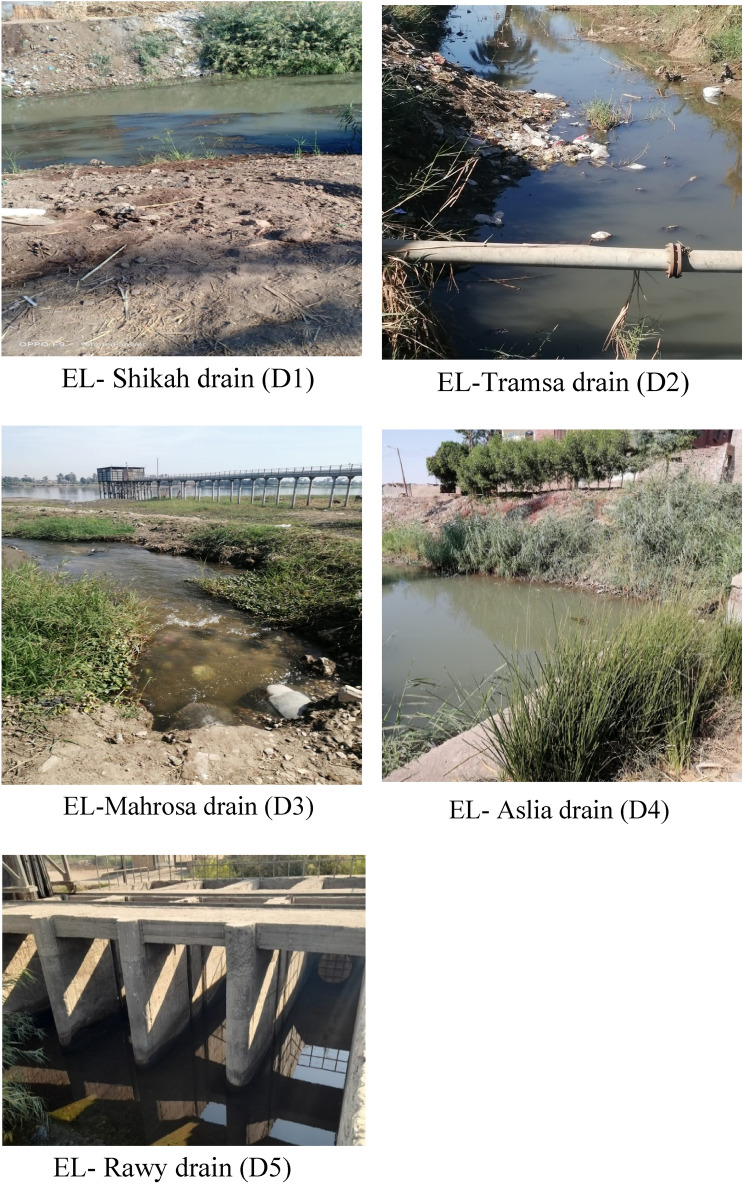
Irrigation drain under study.

Freshwater and sediment samples were collected from 5 irrigation drains (EL-Shikah, EL- Tramsa, EL-Mahrosa, EL-Aslia, and EL-Rawy) located in the geographical area of Qena city, the capital of Qena Governorate, 600 km south of Cairo, (Figs. 1 and 2). 3 sites inside each drain were randomly selected as sampling site; one of these sites represents the outlet of the drain into the Nile River. In addition, one site facing each drain in the main stream of the Nile River was selected to collect freshwater only, thus the total number of samples are 20 freshwater and 15 sediment samples.

Polyethylene Marinelli beakers with a capacity of 1.4 L are used as collection and measuring containers. The beakers were washed with dilute hydrochloric acid and distilled water before use, filled to brim, and then pressed the tight lid to eliminate the internal air. Drops of HNO_3_ were added to the samples to prevent the adhesive of radionuclides with bottle walls^8^.

Sediment samples were collected by Ekman grab sediment sampler. The collected samples were dried using electrical oven at a temperature of 105℃ for 24 h, then sieved through 200 mesh size. The dried samples were filled in hermetical sealed 500 ml polyethylene beakers. The prepared water and sediment samples were stored for 4 weeks to reach a secular equilibrium of radium and thorium with their progenies^9^.

### Measuring systems

Gamma-ray spectrometer consisting of ″3 × 3″ NaI (Tl) detector enclosed in 5 cm thick cylindrical lead shield to reduce the background radiation and connected with 1024 multichannel analyzer was used. The spectrometer was calibrated for energy using ^60^Co and ^137^Cs standard point sources, and calibrated for efficiency using a multi-nuclides standard solution which covers a wide range of energy^10^. The spectrum was accumulated from each sample over 24 h and analyzed by Maestro software. The background was measured under the same condition of sample measurement.^226^Ra was determined using ^214^Bi and ^214^Pb gamma-lines at 609 keV and 352 keV, respectively, while ^232^Th from gamma-lines of ^228^Ac (911 keV) and ^212^Pb (238 keV). ^40^K was determined from its single gamma-line at 1460 keV. The activity concentration was calculated using the following formula (Eq. 1)^11^.

1 $$A = \frac{{C_{n} }}{{T \times \varepsilon { } \times {\text{P}} \times {\text{V }}\left( {{\text{or}}} \right){\text{M}}}}$$

where A is the activity concentration (Bq kg^−1^) or (Bq l^−1^), C_n_ is the net counts under a given peak area, T the sample counting time, $\varepsilon$ is the detection efficiency at measured energy, P is the emission probability and V is the sample volume in liter, M is the sample mass in kilogram. Minimum detectable activity (MDA) was estimated according to Currie definition using Eq. 2^12^ and the MDA values were 0.031, 0.035 and 1.94 Bq L^−1^ for ^226^Ra, ^232^Th, and ^40^K, respectively.

2 $${\text{MDA}} = \frac{2.71 + 465\sqrt B }{{T \times \varepsilon \times P \times V}}$$

where B is the background counts under a given peak area,T,ɛ, P, and V are defined above.

### Doses for aquatic organisms

The external and internal absorbed dose rate for aquatic organisms (Phytoplankton, Mollusca, and Crustacean) in the studied irrigation drains was calculated based on the measured activity concentrations of ^226^Ra, ^232^Th, and ^40^K in environmental media (water and sediment) and using dose conversion coefficients of a given radionuclide for the reference organisms according to the method outlined by Brown et al. described below^13,14^.

3 $$\begin{aligned}& \left( {Sediment\,\, conc. \,\,wet} \right)_{radionuclide} = (Sediment \,\,conc. \,\,dry)_{radionuclide} \times \left( {solids \,\,fraction} \right) \\& \qquad \qquad + (water \,\,conc.)_{radionuclide} \times (1 - \left( {solids \,\,fraction} \right). \\ \end{aligned}$$

4 $$\begin{aligned}& \left( {\user2{External \,\,dose \,\,rate}} \right)_{radionuclide,\, organism} = DPUC_{radionuclide, \,organism}^{external} \times \left[ {Sediment \,conc. \,wet_{radionuclide} \times \left( {fsed_{organism} + fsedsur_{organism} /2} \right)} \right. \\& \quad \quad \left. { + \left( {fwater_{organism} + fsedsur_{organism} /2} \right) \times water \,conc._{radionuclide } /1000} \right] \\ \end{aligned}$$

5 $$\left( {\user2{Internal\,dose\,rate}} \right)_{{radionuclide,\,organism}} = ~\left( {water\,conc.} \right)_{{radionuclide}} \times CF_{{radionuclide}}^{{organism}} \times DPUC_{{radionuclide,\,organism}}^{{internal}}$$

where sediment conc. is the sediment activity concentration of a given radionuclide in Bq kg^−1^,water conc. is the water activity concentration of a given radionuclide in Bq m^−3^, CF is distribution coefficient factors for given radionuclide in freshwater sediment in m^3^ kg^−1^, DPUC is the dose rate per unit concentration coefficients (fresh weight) in *μ*Gy h^−1^ per Bq kg^−1^ weighted for radiation type (alpha = 10, low energy beta = 3, and high energy beta and gamma = 1), solids fraction of wet sediment (0.4), fsed _organism_ is the time fraction spends by organism in sediment, fsedsur _organism_ is the time fraction spends by organism at the sediment/water interface, fwater _organism_ is the time fraction spends by organism in the water column. All parameters used in calculation are taken from Pröhl (2003)^15^ and Vives i Battle et al. (2004)^16^. The total dose is then calculated by summating the external and internal doses.

## Results and discussion

### Natural radioactivity levels in irrigation drains

The average activity concentration of ^226^Ra, ^232^Th, and ^40^K in irrigation water and sediment samples collected from 5 irrigation drains located in Qena governorate, south Egypt are present in Table 1. For irrigation water, the ^226^Ra activity concentration ranged from 0.60 ± 0.05 Bq L^−1^ in EL- Rawy drain to 0.92 ± 0.07 Bq L^−1^ in EL-Tramsa drain with an average value of 0.76 ± 0.06 Bq L^−1^. ^232^Th activity was from 0.21 ± 0.02 Bq L^−1^ in EL-Rawy drain to 0.35 ± 0.03 Bq L^−1^ in EL-Tramsa drain with an average value of 0.27 ± 0.02 Bq L^−1^. ^40^ K activity was from 7.61 ± 0.62 Bq L^−1^ in EL-Shikah drain to 8.67 ± 0.53 Bq L^−1^ in EL-Mahrosa drain with an average value of 8.14 ± 0.71 Bq L^−1^. The results indicate that ^226^Ra, ^232^Th, and ^40^ K activities have a narrow range in the studied irrigation drains, which reflects that the water in these drains comes from the same source, the Nile River. In all studied drains ^226^Ra concentrations are higher than ^232^Th, which may be due to the effect of the fertilizers used for agriculture purposes and the high solubility of radium^17^. ^40^K concentrations are one order of magnitude higher than those of ^226^Ra and ^232^Th, which is consistent with potassium being one of the main elements in nature^18^.

**Table 1:** Average activity concentration of ^226^Ra, ^232^Th, and ^40^ K and ^226^Ra/ ^232^Th ratio in irrigation water and sediment from Qena governorate, Egypt.

Area and code	Irrigation water (Bq L^-1^)				Sediment (Bq kg^-1^)			
	^226^Ra	^232^Th	^40^ K	^226^Ra/^232^Th	^226^Ra	^232^Th	^40^ K	^226^Ra/^232^Th
EL-Shikah (D1)	0.78 ± 0.07	0.30 ± 0.02	7.61 ± 0.62	2.60	21.35 ± 1.22	22.50 ± 1.69	371.65 ± 25.10	0.95
EL- Tramsa (D2)	0.92 ± 0.07	0.35 ± 0.03	8.54 ± 0.92	2.63	30.35 ± 2.10	26.05 ± 1.33	572.10 ± 30.35	1.17
EL-Mahrosa (D3)	0.79 ± 0.06	0.25 ± 0.02	8.67 ± 0.53	3.16	23.91 ± 2.16	15.95 ± 1.05	493.85 ± 42.80	1.50
EL- Aslia (D4)	0.71 ± 0.06	0.26 ± 0.03	8.11 ± 0.75	2.73	19.80 ± 1.69	17.05 ± 1.36	377.10 ± 19.30	1.16
EL- Rawy (D5)	0.60 ± 0.05	0.21 ± 0.02	7.76 ± 0.72	2.86	26.90 ± 2.02	22.05 ± 1.84	450.30 ± 23.15	1.22
All-Average	0.76 ± 0.06	0.27 ± 0.02	8.14 ± 0.71	2.73	24.46 ± 1.84	20.72 ± 1.45	453.00 ± 28.14	1.20

Similar trend was observed in sediment samples, where ^226^Ra concentrations are higher than ^232^Th, and ^40^K concentrations are one order of magnitude higher than both radium and thorium. These results support the fact that sediments act as a sink for the pollutants found in the water column^19^. ^226^Ra values ranged from 19.80 ± 1.69 Bq kg^−1^ in EL- Aslia drain to 30.35 ± 2.10 Bq kg^−1^ in EL-Tramsa drain with an average value of 24.46 ± 1.84 Bq kg^−1^. ^232^Th ranged from 15.95 ± 1.05 Bq kg^−1^ in EL-Mahrosa drain to 26.05 ± 1.33 Bq kg^−1^ in EL-Tramsa drain with an average value of 20.72 ± 1.45 Bq kg^−1^. ^40^ K was from 371.65 ± 25.10 Bq kg^−1^ in EL-Shikah to 572.10 ± 30.35 Bq kg^−1^ in EL-Tramsa drain with an average value of 453.00 ± 28.14 Bq kg^−1^.

The ^226^Ra/^232^Th activity ratios (Table 1) revealed that ^226^Ra activity is on average 2.73 and 1.20 times higher than ^232^Th activity in measured irrigation water and sediment samples, respectively. This could be attributed to the contamination from fertilizers discharge and the solubility and geological differences between ^226^Ra and ^232^Th.

**Figure 3:**
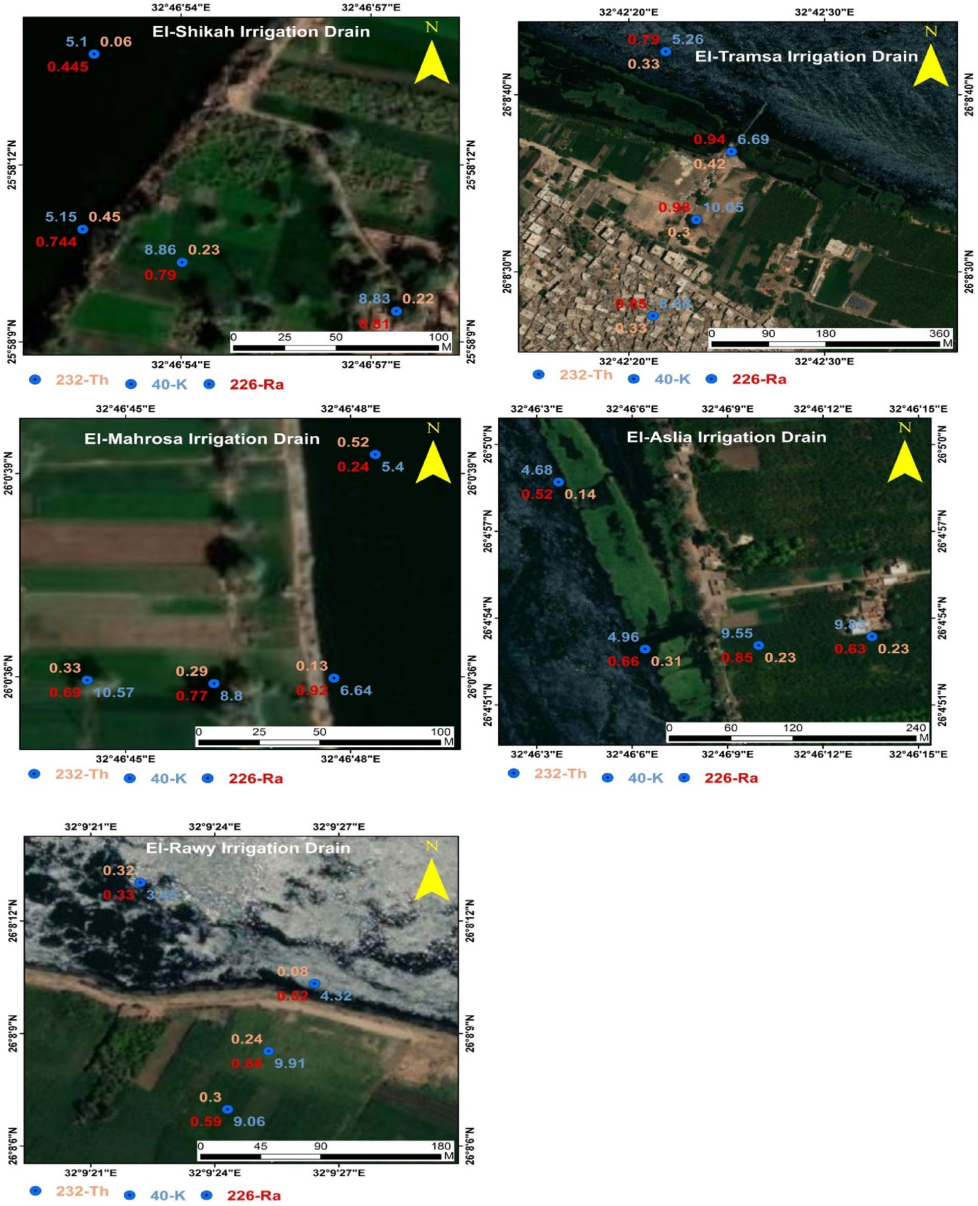
Spatial distribution of natural radionuclides (Bq L^−1^) in the irrigation drains under study **(**(ArcGIS software 10.8.1; ArcGIS Online).

The spatial distribution of ^226^Ra, ^232^Th, and ^40^ K in irrigation water within each is plotted in Fig. 3 using ArcGIS software. It is clear that the radionuclide concentration varies from one place to another within the drain and from one drain to another, which may be due to several factors such as the type and quantity of fertilizer used in the nearby farmlands, the irrigation periodicity of farmlands, and the level of water body of the drain. The figure also shows a lower activity concentration of the studied radionuclides in the mainstream of the Nile river compared to the drains, which reflects the insignificant effect of irrigation water drainage into the Nile on the levels of radionuclides that could be due to the dilution process caused by the large water body of the Nile compared to the drains.

Comparison with previous studies indicated that the activities of ^226^Ra, ^232^Th, and ^40^ K in the current study are higher than those observed for Nasser Lake water (^226^Ra: 0.0033 Bq L^−1^) and Nile water from Assiut, Egypt (^226^Ra:0.20, ^232^Th:0.08, ^40^ K:0.69 Bq L^−1^)^20,21^. Also, sediment samples have higher activities compared to those obtained for Nasser Lake sediment (^226^Ra: 22.0, ^40^ K: 326.20 Bq kg^−1^) and Nile sediment from Qena, Egypt (^226^Ra:14.44, ^232^Th:15.02, ^40^ K:197.57 Bq kg^−1^)^20,22^.

### Dose rates of aquatic organism

**Figure 4:**
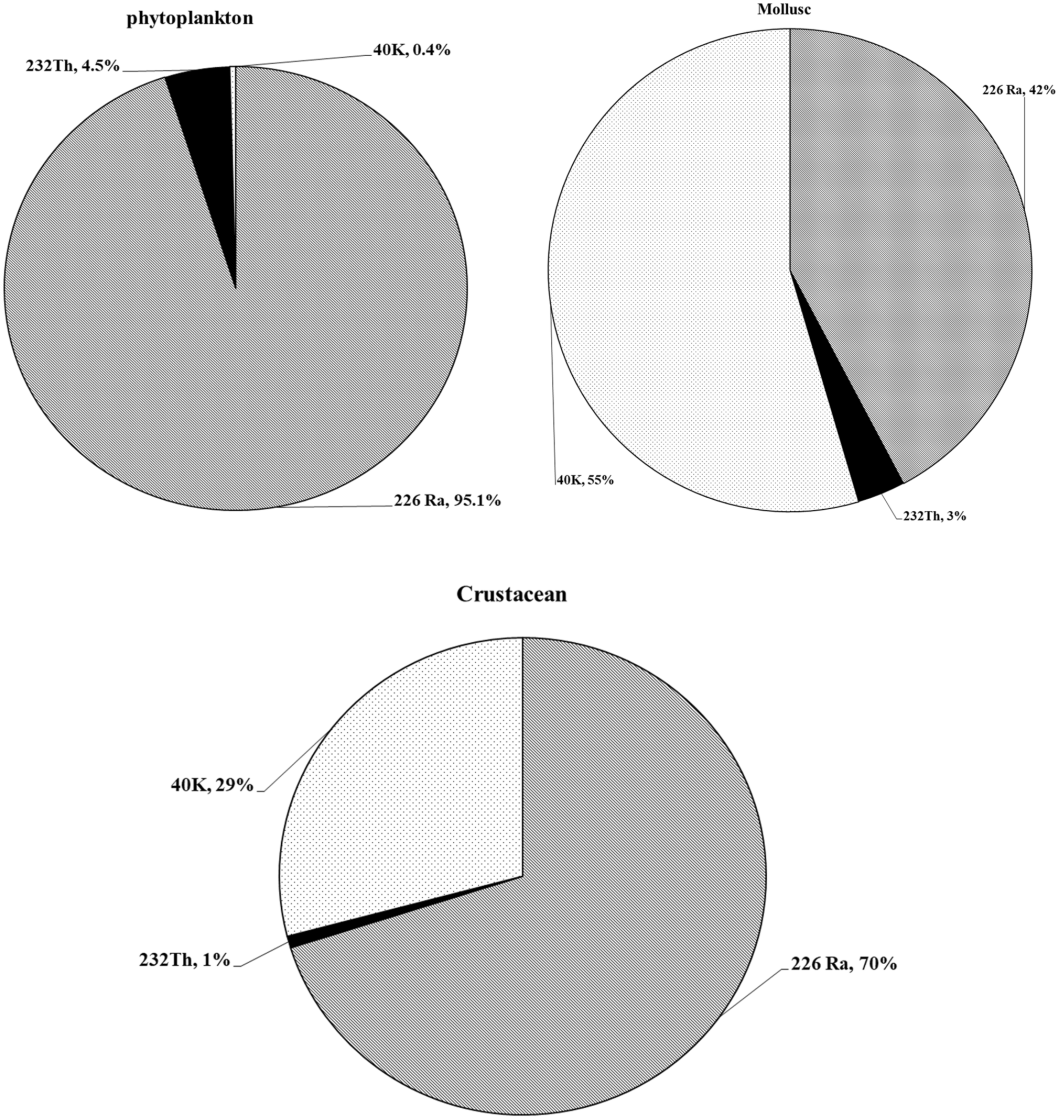
Percentage contribution of natural radionuclides to the internal dose of aquatic organisms.

**Figure 5:**
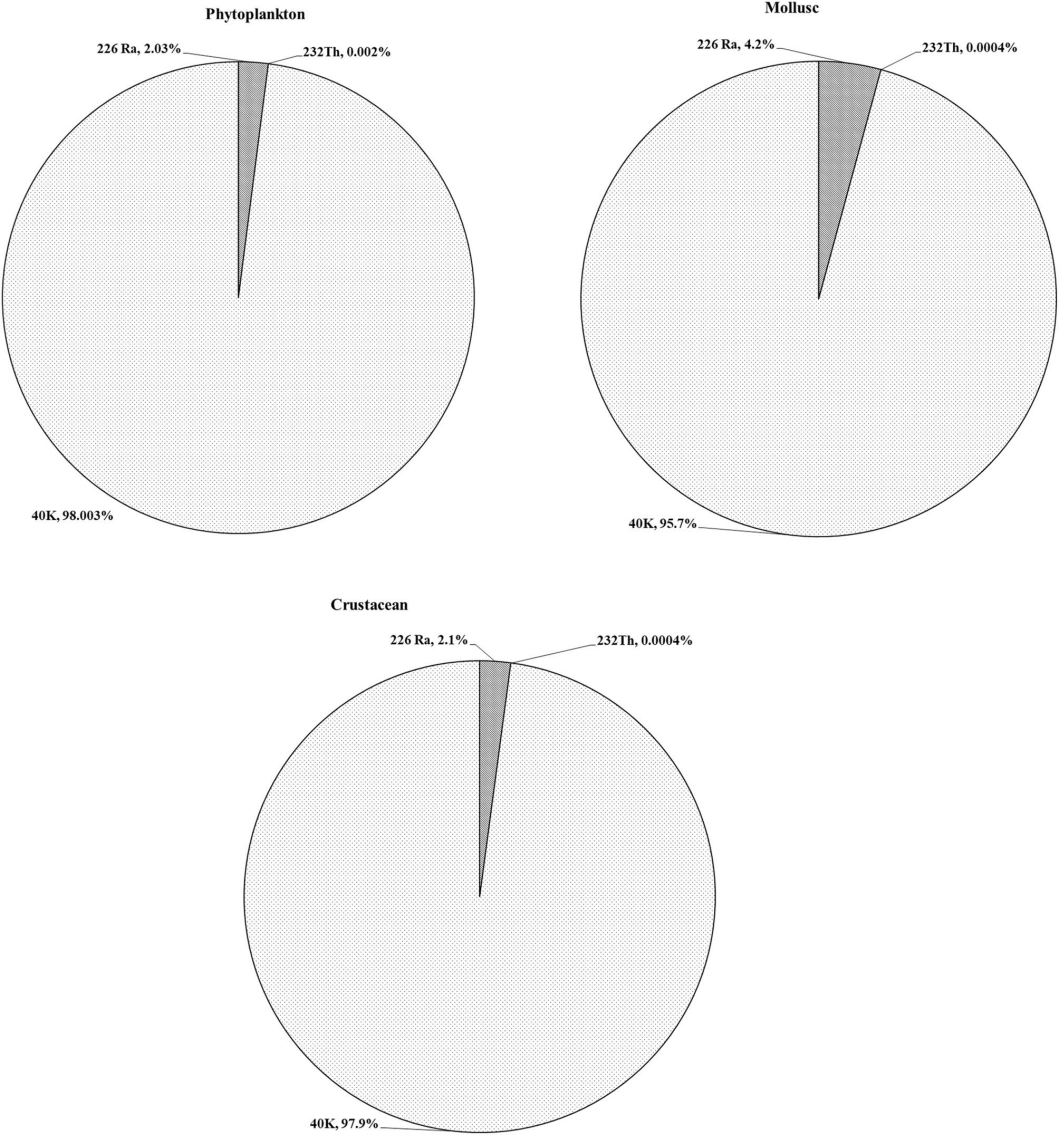
Percentage contribution of natural radionuclides to the external dose of aquatic organisms.

The external, internal, and total absorbed dose rates per aquatic organisms (phytoplankton, mollusc, and crustacean) were calculated according to the procedure outlined above and the average values are present in Table 2. Of the considered aquatic organisms, mollusc received lower doses with values of 1.84 × 10^–04^ and 9.71 × 10^–06^ µGy h^−1^, while phytoplankton received higher doses with values of 7.01 × 10^–04^ and 1.37 × 10^–05^ µGy h^−1^ for external and internal, respectively. Percentage contribution of ^226^Ra, ^232^Th, and ^40^ K to the internal and external doses of the considered aquatic organisms are shown in Figs. 4 and 5. ^226^Ra is the main contributor to the internal dose of phytoplankton and crustacean, while ^40^ K is for mollusc. In mollusc and crustacean, ^232^Th contributes insignificantly to the internal dose, while in phytoplankton the insignificant contribution is from ^40^ K. The external dose received by the considered organisms mainly comes from ^40^ K and negligible contribution comes from ^232^Th and ^226^Ra (Fig. 5). Total dose rates per organism are 7.15 × 10^–04^, 1.94 × 10^–04^, and 5.42 × 10^–04^ µGy h^−1^ for phytoplankton, mollusc, and crustacean, respectively. Comparing these values with the value of 400 µGy h^−1^ recommended by international organizations^4–6^ and the value of 10 µGy h^−1^ recommended by the ERICA tool^23^ for chronic exposure to aquatic organisms, below which it is unlikely that harmful effects will appear. The dose rates of considered organisms in the areas under study are less than the level that is likely to cause harm to aquatic organisms. Therefore, the risks to the aquatic organisms in the area under study are minimal.

**Table 2:** External, internal, and total dose rate per organism in comparison with ERICA dose rate screening value.

Organism name	Dose rate µGy h^−1^		Total dose µGy h^−1^	Screening value* µGy h^−1^
	External	Internal		
Phytoplankton	7.01 × 10^–04^	1.37 × 10^–05^	7.15 × 10^–04^	10.0
Mollusc	1.84 × 10^–04^	9.71 × 10^–06^	1.94 × 10^–04^	10.0
Crustacean	5.22 × 10^–04^	2.00 × 10^–05^	5.42 × 10^–04^	10.0

* ERICA dose rate screening value.

## Conclusions

In this study, the natural radioactivity levels due to ^226^Ra, ^232^Th, and ^40^K were measured by means of gamma spectrometry in the water and sediments of the 5 irrigation drains located in Qena, southern Egypt. The results showed different natural radioactivity levels from one drain to another. In general, the activity levels of ^226^Ra were higher than that of ^232^Th, which reflects the affected of irrigation water by the fertilizers used for agriculture purposes. The activity of ^40^K was one order of magnitude higher than that of ^226^Ra and ^232^Th. By studying the spatial distribution of natural radionuclides of the irrigation water samples in the five drains and their outlets into the Nile river, it was clear that a decrease in the activity level in the Nile mainstream. This may be attributed to the dilution that occurred in the Nile due to the large water body of the Nile compared to drains. Dose rates of aquatic organisms (Phytoplankton, Mollusc, and Crustacean) as a result of exposure to natural radionuclides ^226^Ra, ^232^Th, and ^40^K were calculated, and the results showed that it is unlikely that harmful effects will appear on those organisms due to exposure to the considered radionuclides in the environment under study. This study provides the first basic data on radioactivity levels in the freshwater environment in Egypt and assesses the associated radiological risks to aquatic organisms. Given the importance of this type of study, regular monitoring of the levels of natural and artificial radioactivity in those environments is important for the assessment of radiological risks in order to protect those organisms and the environment in general.

## Data Availability

The data used for this study will be available from the corresponding author upon request.
